# Photovoltage oscillations in encapsulated graphene

**DOI:** 10.1038/s41598-022-09025-y

**Published:** 2022-03-25

**Authors:** Jesús Iñarrea, Gloria Platero

**Affiliations:** 1 Escuela Politécnica Superior, Universidad Carlos III, 28911 Leganés, Madrid Spain; 2 Instituto de Ciencia de Materiales, CSIC, Cantoblanco, 28049 Madrid, Spain; 3 Unidad Asociada al Instituto de Ciencia de Materiales, CSIC, Cantoblanco, 28049 Madrid, Spain

**Keywords:** Materials science, Nanoscience and technology, Physics

## Abstract

We theoretically analyze the rise of photovoltage oscillations in hexagonal boron-nitride (h-BN) encapsulated monolayer graphene (h-BN/graphene/h-BN) when irradiated with terahertz radiation. We use an extension of the radiation-driven electron orbit model, successfully applied to study the oscillations obtained in irradiated magnetotransport of GaAs/AlGaAs heterostructures. The extension takes mainly into account that now the carriers are *massive* Dirac fermions. Our simulations reveal that the photovoltage in these graphene systems presents important oscillations similar to the ones of irradiated magnetoresistance in semiconductor platforms but in the terahertz range. We also obtain that these oscillations are clearly affected by the voltages applied to the sandwiched graphene: a vertical gate voltage between the two hBN layers and an external positive voltage applied to one of the sample sides. The former steers the carrier effective mass and the latter the photovoltage intensity and the oscillations amplitude. The frequency dependence of the photo-oscillations is also investigated.

## Introduction

Microwave-induced resistance oscillations (MIRO)^1,2^, predicted by Ryzhii^3,4^ in the 70’s, were discovered two decades ago in high mobility two dimensional electron systems (2DES) (GaAs/AlGaAs heterostructure) when irradiated under a vertical magnetic field (*B*) at very low temperatures, $T \sim 1\;\text{K}$. Most of the experimental^5–22^ and theoretical^23–39^ works since then, have been focused on the effect of radiation on the magnetoresistance ($R_{xx}$) of the sample. Nevertheless, other measurable physical quantities could be of interest to study photo-oscillations in 2D systems such as, for instance, the photovoltage. The interest rises if instead of 2D Schrödinger electrons we use the paradigmatic 2D platform nowadays, i.e., monolayer graphene or other graphene structures^40^. In this way, an experimental work^41^ has been recently published probing the rise of MIRO-like photovoltage oscillations in h-BN encapsulated graphene keeping most of MIRO characteristics. On the other hand, a previous and pioneering theoretical work predicting radiation-induced resistance oscillations in monolayer and bilayer graphene^42^ was reported.

In this letter, we present theoretical results on the presence of MIRO-like magnetooscillations in the photovoltage of the trilayer system of h-BN encapsulated monolayer graphene. In this system the carriers are *massive* Dirac fermions^43–48^ presenting one of the highest mobilities ($\mu \sim 3 \times 10^{5}$ $\text{cm}^{2}/\text{Vs}$) among the graphene systems. Here we extend the theoretical model of *radiation-driven electron orbits*^23,24^ previously applied to 2D semiconductor heterostructures to address MIRO and zero resistance states (ZRS). In our simulations we first obtain that hBN-encapsulated graphene is mostly sensitive to terahertz (THz) radiation instead of microwaves (MW). We study the dependence of the photovoltage oscillations with respect to a vertical gate voltage which is able to tune the electron mass and in turn the oscillations position with respect to the magnetic field. Next, and keeping constant the gate voltage, we study the influence of an extra external voltage on the oscillations position and intensity. We obtain that we can increase the oscillations amplitude just by raising the external voltage without changing the radiation power. We have investigated also the photovoltage dependence with radiation frequency in the terahertz (TH) range. The results presented in this letter would be of special interest from the application perspective, for instance in nanophotonics; they could lead to the design of novel ultrasensitive terahertz detectors or to the proposal of a new generation of solar cells given the strong translation of radiation energy into electrical current.

## Theoretical model

When monolayer graphene is placed on top of a substrate or encapsulated, the interaction between the layers gives rise to a potentials asymmetry between the graphene sublattices A and B^49,50^. In the same way, a vertical gate voltage can produce this asymmetry which, in this case, can be externally tuned. As a result a band gap opens at the Dirac point (charge neutrality point) giving rise to gapped monolayer graphene. Thus, monolayer graphene turns from semimetal into a semiconductor and the Dirac fermions become massive. The eigenenergies and eigenfunctions of the gapped graphene Hamiltonian in the presence of an external and perpendicular magnetic field, can be readily calculated^51,52^, for instance for the *K* valley:

1 $$\begin{aligned} E_{n,K}= & {} \pm \sqrt{(\hbar w_{B})^{2} |n| +\Delta ^{2}}; (n=\pm 1,\pm 2,....)\end{aligned}$$

2 $$\begin{aligned} E_{0,K}= & {} -\Delta \end{aligned}$$

for the energies and

3 $$\begin{aligned} \Phi _{n,K} \propto \left( \begin{array} {cc} \phi _{|n|-1}\left( x+\frac{\hbar k_{y}}{e B}\right) \\ \phi _{|n|}\left( x+\frac{\hbar k_{y}}{e B}\right) \end{array} \right) \end{aligned}$$

for the eigenstates, where $\phi _{n}$ is the standard Landau level wave function (Landau state), $w_{B}=v_{F}\sqrt{2}/l_{B}$, $l_{B}$ being the magnetic length and $v_{F}$ the Fermi velocity. $\Delta$ is a massive term that represents the potential asymmetry between the two sites, A and B.

An important point in this scenario is that close to the conduction band bottom in the gapped monolayer graphene, the Dirac fermions are A-sublattice-polarised^51,52^. On the other hand, close to the valence band top, the amplitude of the wave function is predominant at the B sublattice (B-sublattice polarised). Thus, around the conduction band bottom we can approximately express the eigenstates at the *K* valley as,

4 $$\begin{aligned} \Phi _{n,K} \propto \left( \begin{array} {cc} \phi _{|n|-1}\left( x+\frac{\hbar k_{y}}{e B}\right) \\ \sim 0 \end{array} \right) \end{aligned}$$

If we expand the gapped graphene Hamiltonian^51^, for instance, near the conduction band bottom we can obtain an effective expression^52,53^

5 $$\begin{aligned} \left[ \frac{P_{x}^{2}}{2m^{*}}+\frac{(P_{y}^{2}+eBx)}{2m^{*}}\mp \frac{1}{2}\hbar w_{c}\right] \Phi _{n,K/K'} = E_{n,K/K'}\Phi _{n,K/K'} \end{aligned}$$

where the cyclotron frequency $w_{c}=eB/m^{\star }$, $m^{*}$ being the effective mass of the corresponding massive Dirac fermion, $m^{*}=\frac{\Delta }{v_{F}^{2}}$. The gap energy is twice $\Delta$. Thus, $m^{*}$ turns out to be gap dependent and can be tuned, for instance by an external bias. In Eq. (5) the ‘−’ sign would correspond to *K* and ‘+’ to $K'$ valley.

To study magnetotransport in encapsulated graphene under radiation and *B*, we follow the previously developed *radiation-driven electron orbits model*^23,36^ starting from the Hamiltonian in Eq. (5). Accordingly, the time dependent Hamiltonian can be exactly solved^23,24,36,55^ allowing a solution for the massive Dirac fermion wave function (for the *K* valley):

6 $$\begin{aligned} \Phi _{n,K} \propto \left( \begin{array} {cc} \phi _{|n-1|}\left( x+\frac{\hbar k_{y}}{e B}-x_{cl}(t),t)\right) \\ \sim 0 \end{array} \right) \end{aligned}$$

The time-dependent guiding center shift $x_{cl}(t)$ is given by:

7 $$\begin{aligned} x_{cl}(t)= & {} \frac{e^{- \gamma t/2} e E_{o}}{m^{*}\sqrt{(w_{c}^{2}-w^{2})^{2}+\gamma ^{4}}}\sin wt\nonumber \\= & {} A(t)\sin wt \end{aligned}$$

where $E_{0}$ is the radiation electric field amplitud and *w* the frequency. Thus, the radiation-driven Landau states, perform a swinging motion where the massive Dirac fermions interact with the lattice ions resulting in a damping process of the oscillating classical motion. The corresponding oscillation damping is phenomenologically introduced through the $\gamma$-dependent damping term^53^.

**Figure 1:**
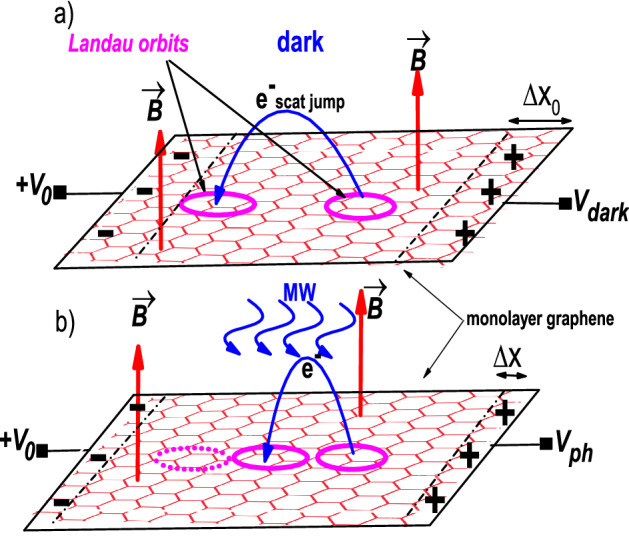
Schematic diagram showing the dynamics of dark and irradiated monolayer graphene (encapsulated) under external voltage $+V_{0}$. (**a**) In the dark case the scattered electron mostly jump, between Landau orbits, in the direction of the positive voltage. As a result two lines of opposite charges build up at facing sides of the sample. (**b**) With radiation, the scattering jump distance changes harmonically with *B* giving rise to radiation-induced photovoltage oscillations.

According to our previous theoretical model, the MIRO-like oscillations obtained when measuring irradiated magnetoresistance^53^ vs *B* are a direct consequence of an interplay between the driven-swinging motion of the Landau orbits and the electron scattering with charged impurities. This harmonic motion performed by the Landau orbits can be revealed by measuring photovoltage on encapsulated graphene instead of the usual magnetoresistance^53^. Then, the corresponding oscillations are expected to be obtained. In our theoretical approach we first consider that one of the squared sample edges is connected to an external positive DC voltage, $+V_{0}$ (see Fig. 1). As a result the electron scattering between Landau orbits shows, instead of random, a predominant direction, the one of the positive external voltage. This gives rise on average to an asymmetric distribution of charge on the sample, similar to a charge displacement in the scattering (transport) direction. Then, two lines of opposite charge at either side of the sample show up (see Fig. 1) of a width of the order of the spatial scattering jump between orbits, $\Delta X_{0}$^23,24,36^. A subsequent electric field and voltage drop, $V_{dark}$, along the sample is then created and can be experimentally measured. $V_{dark}$ can be straightforward calculated from basic electrostatics in terms of the 2D electron (charge) density per unit area $n_{2D}$ and the displacement $\Delta X_{0}$,

8 $$\begin{aligned} V_{dark}=\frac{n_{2D} e \Delta X_{0}}{2\pi \epsilon } \end{aligned}$$

where *e* is the electron charge, $\epsilon$ is the graphene permittivity and $n_{2D} e \Delta X_{0}$ is the charge density in the lines. $n_{2D}$ can be expressed in function of the density of states per unit area and energy *D*(*E*). We focus on the states around the Fermi energy; electrons occupying these states will mainly take part in the scattering. Thus, $n_{2D}(E_{F})= 2 D(E_{F}) \Delta E_{F}$, where $\Delta E_{F}$ is an energy interval around the Fermi energy. The density of states is given by^54^:

9 $$\begin{aligned} D(E_{F})=\frac{2m^{*}}{\pi \hbar ^{2}}\left[ 1+\frac{2X_{s}}{\sinh X_{s}}e^{-\frac{\pi \Gamma }{\hbar w_{c}}} \cos 2\pi \left( \frac{E_{F}}{\hbar w_{c}}+\frac{1}{2}\right) \right] \end{aligned}$$

Thus, $D(E_{F})$ accounts for the SdH oscillations that show up in the photovoltage. $\Gamma$ is the Landau state width and $X_{s}=\frac{2\pi ^{2}K_{B}T}{\hbar w_{c}}$, $K_{B}$ being the Boltzman constant.

Following our model, when irradiated the Landau orbits begin to harmonically swing giving rise to shorter and longer scattering jumps depending on the magnetic field (see Fig. 1). Then, the spatial shift $\Delta X_{0}$ turns into a harmonic function^55^ $\Delta X=\Delta X_{0}-A \sin \left( 2\pi \frac{w}{w_{c}}\right)$ and instead of $V_{dark}$ there is a photovoltage, $V_{ph}$ given by an expression similar to $V_{dark}$ but with $\Delta X$ instead of $\Delta X_{0}$. Therefore, in the two kinds of oscillations obtained in the irradiated photovoltage, the SdH oscillations are explained with the density of states and the MIRO-like ones are due to $\Delta X$. The former would be obtained irrespective of the presence of radiation but the latter are only seen with radiation.

## Results

**Figure 2:**
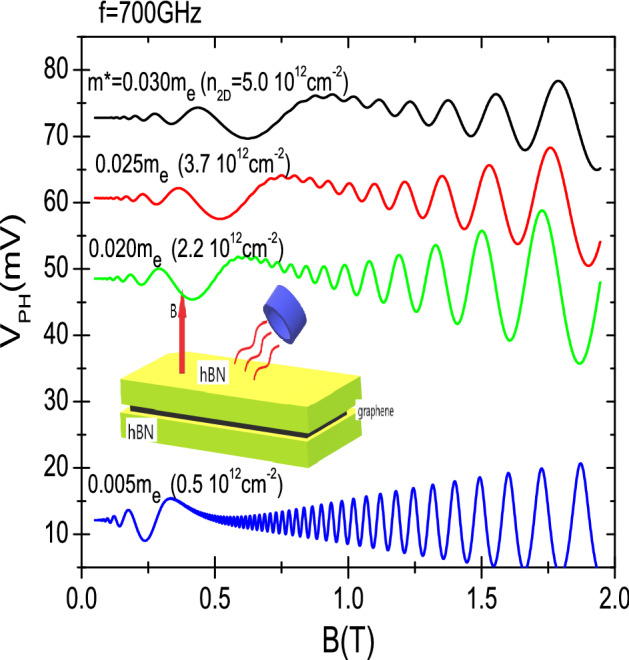
Calculated photovoltage, $V_{ph}$, under radiation vs magnetic field for h-BN encapsulated monolayer graphene. The radiation frequency is 700 GHz and temperature $T=1\;\text{K}$. Every effective mass (charge density) corresponds to a different bias between the hBN layers (top and bottom). We observe that the oscillations amplitude does not significantly change with the effective mass (gate voltage). The inset shows a schematic diagram of the h-BN sandwiched monolayer graphene under a constant magnetic field and radiation.

In Fig. 2 we exhibit irradiated photovoltage vs *B* for a gated hBN-encapsulated monolayer graphene. The radiation frequency is 700 GHz and $T=1.0 \; \text{K}$. The inset shows the basic experimental set up: irradiated encapsulated monolayer graphene under *B*. The hBN/graphene/hBN sandwich yields a bandgap in the monolayer graphene beeing tuned by a gate voltage between the two outer hBN layers^56^. This allows us to select the Dirac fermions effective mass just by tuning the gate voltage. With the latter we can tune as well the charge density (Fermi level) in the monolayer graphene. Thus both, effective mass and charge density, can be simultaneously altered by a varying vertical electric field (gate voltage). Therefore, for a definite gate voltage the system has a definite band gap and charge density. Based on a previous work^56^, the electric field we have used in the simulations ranges from 0.10 to 1 V/${\text{\AA} }$ that correspond to band gaps from 90 meV to 340 meV. In turn, the effective mass, $m^{*}=\Delta /v_{F}^{2}$, goes from $0.005 m_{e}$ to $0.030m_{e}$, where $m_{e}$ is the bare electron mass. With these low values of $m^{*}$ it is expected these systems to be sensitive to higher radiation frequencies. In this way, an important result of the simulations is that clear photovoltage oscillations with all MIRO features turn up at THz radiation frequencies and low *B*. In previous simulations it was found that irradiated magnetoresistance (instead of photovoltage) of sandwiched hBN graphene presented MIRO-like oscillations at THz frequencies too^53^. In order to contrast with experiment^41^ we can obtain approximate values of $n_{2D}$ in terms of the above electric fields^56^ adapted to the sample thickness of experiment^41^. Our calculations, based on basic electrostatics, yields a charge density $n_{2D}=\frac{\epsilon E_{\perp }}{e}$, that goes from $0.5 \times 10^{12}$ $\text{cm}^{-2}$ to $5. \times 10^{12}$ $\text{cm}^{-2}$, $E_{\perp }$ being the vertical electric field. These charge density values are quantitatively similar (same order of magnitude) to the ones of experiment^41^. Then, each curve exhibited in Fig. 2 is labelled with both, effective mass and charge density. The results presented in Fig. 2 corresponds to a scenario of constant external voltage, $+V_{0}$ and varying gate voltage (effective mass and charge density). We observe that as $m^{*}$, the photovoltage curve lowers too. This is due to that $m^{*}$ enters directly in the expression of $D(E_{F})$ and in turn to $V_{ph}$. Nevertheless, the relative size of the oscillations amplitude keeps approximately constant and remains unaffected by the changing $m^{*}$. In this case $m^{*}$ shows up also in the denominator of *A* keeping its magnitude approximately constant irrespective of $m^{*}$. On the other hand, the irradiated oscillations move to lower *B* as $m^{*}$ decreases because it makes decrease the oscillations frequency. The latter is a basic features of standard MIRO.

**Figure 3:**
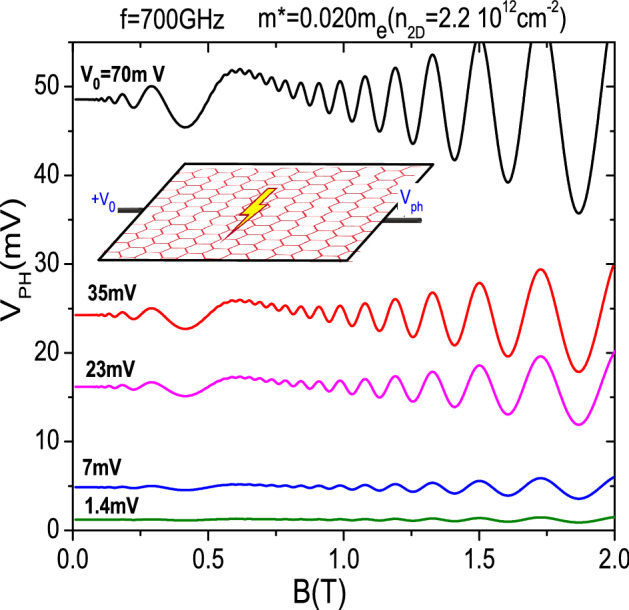
Calculated photovoltage vs *B* for different values of the external positive voltage, $+V_{0}$. The gate voltage is kept constant giving a $m^{*}=0.020m_{e}$. The radiation frequency is 700 GHz and $T=1\;\text{K}$. From the upper to the lower curve the external voltage is gradually decreased. For the lowest value the oscillations, both MIRO-like and SdH, are practically washed out.

In Fig. 3 we exhibit irradiated photovoltage vs *B* for an opposite scenario to Fig. 2, i.e., we represent different values of the external positive voltage, $V_{0}$ whereas the gate voltage is kept constant giving a $m^{*}=0.020m_{e}$. The radiation frequency is 700 GHz and $T=1\;\text{K}$. From the upper curve to lower the external voltage is gradually decreased and for the lowest value the oscillations, both MIRO-like and SdH, are practically wiped out. $V_{0}$ is directly responsible of the appearance of the two lines of charge and in turn directly proportional to the amount of charge; the larger (smaller) $V_{0}$ the larger (smaller) the charge accumulated in the lines. Thus, keeping $m^{*}$ constant, a decreasing $V_{0}$ leads to the disappearance of the lines of charge. Then, the whole curve goes to zero as $V_{0}$ approaches zero too and a flat line would be obtained for the photovoltage vs *B* instead. This result has been experimentally obtained in GaAs/AlAs platforms under MW and THz^57^. In contrast to the previous figure, where a varying gate voltage made change the MIRO-like oscillations positions, here the varying external voltage does not affected them at all. They keep the same positions irrespective of $V_{0}$. According to these simulations, when the external voltage is zero the whole photovoltage would be zero too irrespective of the frequency and intensity of the incoming radiation. Nevertheless we would obtain some response around the cyclotron resonance but MIRO and SdH oscillations would not show up. Thus, an important conclusion is that we need an external voltage to be applied in order to obtain those oscillations. On the other hand, large amplitude irradiated oscillations would require large values of $V_{0}$. Then, an remarkable consequence, from the application standpoint, is that we can tune the amplitude of the radiation response just by varying the external voltage independently of the radiation intensity. Thus, increasing enough the external voltage $V_{0}$, we would obtain a clear electrical response for radiation of very low intensity acting the graphene device as a signal amplifier, for instance in the THz range. This could have interesting implications from the application side.

**Figure 4:**
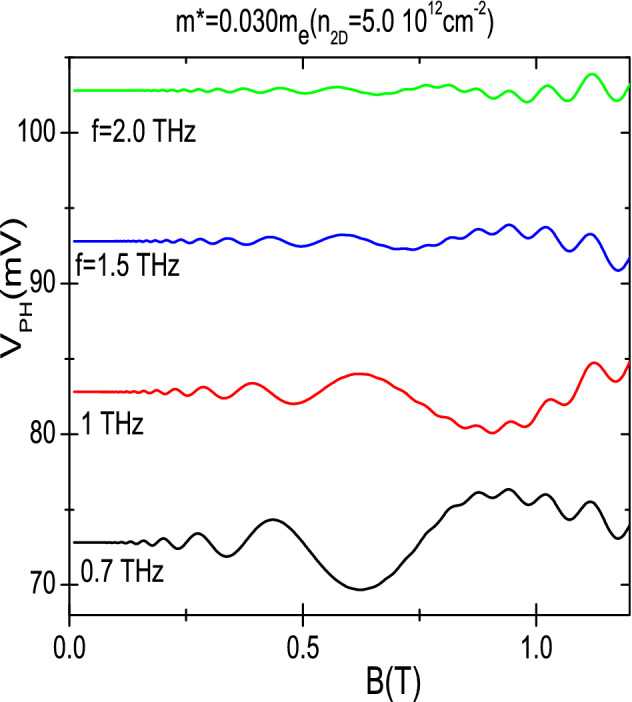
Frequency dependence of the calculated photovoltage vs *B*, showing four frequencies in the THz range: 0.7, 1, 1.5 and 2.0 THz. As the frequency rises the number of oscillations increases but the oscillations amplitude lowers. $T=1$ K.

In Fig. 4 we exhibit the frequency dependence of calculated photovoltage vs *B*. Four different frequencies in the THz range are presented: 0.7, 1, 1.5 and 2.0 THz. We observe that as the frequency rises the number of oscillations increases too but the oscillations amplitude lowers. The former turns out obvious and the latter is expected according to our theory as the frequency shows up in the denominator of the amplitude. These results agree with experimental results^41^ and with the frequency dependence with standard MIRO obtained in semiconductor platforms and MW. More simulations have been run at higher frequencies confirming the trends in terms of number of oscillations and decreasing amplitude.

## Conclusions

Summing up, we have presented a theoretical approach on the appearance of photovoltage oscillations in h-BN encapsulated monolayer graphene irradiated with terahertz radiation. In our model we have extended the radiation-driven electron orbit model to the case of massive Dirac fermions. Our simulations have revealed that the photovoltage in these graphene systems presents important oscillations in the terahertz range. In the same way, these oscillations are clearly affected by the different voltages applied to the sandwiched graphene; mainly, a vertical gate voltage between the two hBN layers that steers the effective mass and an external positive voltage that affects the photovoltage intensity and the oscillations amplitude. We have also studied the frequency dependence on photovoltage obtaining similar results as in previous MIRO, i.e., when the frequency increases the number of oscillations increases and the amplitude lessens. The results above would be of interest from the application point of view in any field where the radiation-matter interaction is key being based on graphene. For instance, novel ultrasensitive terahertz detectors or sensors, a new generation of solar cells given the strong translation of radiation energy into electrical current, etc.
